# The institutional primary healthcare service quality and patients’ experiences in Chinese community health centres: results from the Greater Bay Area study, China

**DOI:** 10.1186/s12939-021-01538-8

**Published:** 2021-08-30

**Authors:** RuQing Liu, Leiyu Shi, YiFan Meng, Ning He, JingLan Wu, XinWen Yan, RuWei Hu

**Affiliations:** 1 Guangdong Provincial Engineering Technology Research Center of Environmental Pollution and Health Risk Assessment, Department of Occupational and Environmental Health, School of Public Health, Sun Yat-Sen University, 510080 Guangzhou, China; 2 Department of Health Policy & Management, John Hopkins School of Public Health, Baltimore, MD 21205 USA; 3 Department of Health Management, School of Public Health, Sun Yat-Sen University, 74 Zhongshan 2nd Road, Yuexiu District, Guangzhou, 510080 China; 4 Johns Hopkins University, Baltimore, MD 21218 USA

**Keywords:** Community health centres, National Committee for Quality Assurance Patient-Centred Medical Home, Primary care assessment tools

## Abstract

**Background:**

The goal of this paper was to assess the quality of primary healthcare services at community health centres (CHCs) from the demand (patient) and supplier (healthcare service institution) angles.

**Methods:**

This study was conducted at six CHCs in the Greater Bay Area of China. Between August and October 2019, 1,568 patients were recruited (55.8% women and 44.2% men). We evaluated the service quality of CHCs using the National Committee for Quality Assurance Patient-Centred Medical Home (NCQA-PCMH) recognition questionnaire. We assessed patients’ experiences with medical and health services using the Primary Care Assessment Tools (PCAT).

**Results:**

PCAT total and sub-domains scores were significantly difference at the six CHCs (*P* < 0.001). Among the six CHCs, Shayuan CHC had the highest PCAT total and sub-domain scores and the highest NCQA-PCMH total and sub-domain scores, as well. Older (> 60 years), female, lower education, and employee medical-insured individuals had better patient experiences.

**Conclusions:**

Our results indicate that CHCs could improve their service quality by improving both institutional health service quality based on NCQA-PCMH assessment and patient experiences based on PCAT scales. These findings can help inform patient-centred primary healthcare policy and management.

**Supplementary Information:**

The online version contains supplementary material available at 10.1186/s12939-021-01538-8.

## Introduction

The outbreak of the 2019 novel coronavirus (COVID-19) has evolved into a global crisis. As the frontline of defence against pandemic prevention and control, community health centres (CHCs) play important roles in primary health screening and management globally [1–3]. Thus, enhancing the service quality of CHCs to effectively deal with health emergencies and community health management is of high priority. In order to effectively reform CHC service quality, however, data must first be collected to evaluate the current service quality status.

Quality evaluation can be defined by three dimensions: ‘structure–process–outcome,’ with better process quality leading to better outcome quality, according to Donabedian, the father of quality management in the United States [4]. To determine the overall quality (service quality) of primary healthcare, process quality can be determined using the institution as the supplier and outcome quality can be determined using patients as the demanders.

Some studies have tried to assess primary healthcare service quality from the demand (patients) and supply (healthcare service institution) sides to explore the association between the institutional primary healthcare service quality and patients’ experiences [5–8]. However, few of these studies assessed primary healthcare service quality from the institutional side. Even so, the existing results have been inconsistent, perhaps due to the inherent subjectivity of how patients perceive quality. Starfield and his colleagues analysed the differences between consumers’ experiences and provider reports in four main domains of primary care (first contact, ongoing care, comprehensiveness, and coordination) and three related domains (family centredness, community orientation, and culturally competent care). There were no significant differences between consumers and providers in perceived quality of primary care received or provided [5]. However, other studies found an association between the evaluation of primary healthcare quality services determined by patients and health institutions. The results from a Spanish study indicated that the evaluation of service quality among staff was higher than that among patients with the Primary Care Assessment Tool (PCAT), which is used as a quantitative assessment tool for patient evaluation [6]. In China, according to a quantitative study using PCAT, patients evaluated first-level hospitals more favourably than second- and third-level hospitals [7]. Our previous study, also using PCAT, indicated that CHCs provide better primary healthcare services than secondary and tertiary health institutions in China, even after controlling for sociodemographic and medical characteristics [8]. Different evaluation standards of service quality, various assessment scales, and differences in population characteristics may have contributed to the heterogeneity of the published results. Furthermore, the most obvious weakness of existing studies is the lack of quantitative evaluation of institutional primary healthcare service quality. Thus, a need remains for additional investigation to assess the primary healthcare service quality from the demand (patients) and supplier (healthcare service institution) sides, especially with quantitative institutional and patient evaluations.

The National Committee for Quality Assurance Patient-Centred Medical Home (NCQA-PCMH) is officially recognised as a quantitative assessment tool of institutional evaluation (supply-side) [9], and the PCAT is widely used as a quantitative assessment tool for patient evaluation (demand-side) [10]. Using both the NCQA-PCMH and the PCAT in assessing service quality may provide more reliable insights when evaluating CHCs. However, this dual assessment method has not been used globally. To address pending data gaps, we assessed the institutional service quality of CHCs determined by NCQA-PCMH and the patient experiences of CHCs as determined by the PCAT at the same time. We also tried to figure out what demographic characteristics could influence the PCAT scores among the patients, and then to give the hints to the CHCs to improve their service quality.

## Methods

### Study area and study population

This study was conducted in select urban CHCs in Guangzhou, the biggest metropolis in the Greater Bay Area, southern China. We employed a multi-stage, stratified clustering sampling protocol. Data were collected from August to October 2019. In our sampling strategy, the area was stratified into four urban districts: Liwan, Yuexiu, Tianhe, and Haizhu. Two urban CHCs were randomly selected from each of Tianhe and Haizhu districts, respectively, and one urban CHC was selected from each of Yuexiu and Liwan districts, respectively. As shown in Figure S1, six CHCs were randomly selected: Linhua (LH), Liede (LD), Jianghai (JH), Shayuan (SY), HuangHuagang (HHG), and Hualin (HL). Then, one family physician group was selected randomly from each selected CHC. In the last sampling stage, participants were randomly recruited by the selected family physician group during their visit to CHCs. Finally, one participant aged 20 years or older without audio or visual impairment, mental illness, or other issues that might interfere with study participation that had been a resident of the district for at least 1 year was recruited.

Written informed consent from each participant was obtained prior to data and sample collection. The study procedure was approved by the Human Studies Committee of Sun Yat-sen University in compliance with the Declaration of Helsinki—Ethical Principles for Medical Research Involving Human Subjects (no. IRB2014.9).

### Evaluation of service quality of institutions

We used the NCQA-PCMH recognition questionnaire to evaluate the institutional primary healthcare service as the supplier. The National Committee for Quality Assurance Patient-Centred Medical Home (NCQA-PCMH) has been officially recognised as an institutional primary healthcare service quality assessment tool [9]. The NCQA-PCMH is ‘the most economic and suitable’ healthcare service model according to the World Health Organization and was developed in the United States [9]. As of 2020, about 13,000 primary care institutions and 67,000 physicians in the United States had been accredited so that they could identify service quality problems to improve patient satisfaction and reduce healthcare costs according to the results from NCQA-PCMH assessments [11–13]. The 2014 version of the NCQA-PCMH survey tool was authorised by the American NCQA website [11] and translated into Chinese. The 2014 version of the NCQA-PCMH scale included six sections: PCMH1, patient-centred access; PCMH2, team-based care; PCMH3, population health management; PCMH4, care management and support; PCMH5, care coordination and care transitions; and PCMH6, performance measurement and quality improvement. Each section had 3–7 evaluation elements, including a necessary element (for a total of 27 elements), and 2–11 specific items for each element, with each including a key entry (for a total of 178 items). Every element in each section had separate scoring criteria and rules. According to the compliance of the criteria required by the evaluation agency, each element is scored by a percentage (if it does not meet the key entry of the element, the individual percentage of the element is 0%). The total score is calculated according to the scores for each element and section. The score is capped at a maximum of 100, meaning institutions can be divided into three levels by their final score: 35–59 for Level 1, 60–84 for Level 2, and 85–100 for Level 3 [14]. The higher the NCQA-PCMH level of the organisation, the better the service quality and the lower the service cost [11]. Therefore, patients favour higher scoring organizations and can implement medical insurance purchase decisions based on the results of cost-quality assessments [14].

### Assessment of patients’ experiences

We used the consumer-client version of the PCAT to identify the quality of primary care services for evaluating the demand-side. The Primary Care Assessment Tool (PCAT) is a series of scales that have been developed by the Primary Care Policy Centre of Johns Hopkins University, which evaluate the primary healthcare service quality based on patients’ experiences [10]. The PCAT was originally validated by the Johns Hopkins Primary Care Policy Centre in the United States to measure the quality of primary care services [5]. Unlike other questionnaires that focus on subjective satisfaction, the PCAT focuses on real experiences of patients obtaining primary care services in CHCs. Thus, the results of the PCAT are more objective [10]. We used an unmodified Chinese language version of the original simplified PCAT scale in the present study. The PCAT includes the following 11 dimensions related to the core primary care domains: A. Extent of affiliation with a place/doctor, B. First contact in terms of utilisation (the extent to which the primary care provider performs a gatekeeper function), C. First-contact care in terms of access (whether patients can contact a physician in time when they need medical and health services), D. Ongoing care (the continuous relationship between physicians and patients in primary care institutions), E. Coordination of care (the interpersonal linkage of care among different levels of providers), F. Coordination of information systems (informational linkage of care through the use of an electronic information system), G. Comprehensiveness of services available (the ability to perform a wide range of services in primary care), H. Comprehensiveness of services provided (the appropriate provision of services during consultations by a primary care provider), I. Family centredness (the recognition of the family as a major participant in the diagnosis, treatment, and recovery of patients), J. Community orientation (whether CHCs fully consider the needs of patients in the implementation of health services), and K. Culturally competent care (the provision of care that respects the beliefs, interpersonal styles, attitudes and behaviors of people as they influence health). Each dimension contains 3–5 items. All items in the PCAT were represented by a 4-point Likert-type scale (1 = *definitely not*, 2 = *probably not*, 3 = *probably*, and 4 = *definitely*). The average score for each scale was derived by averaging the values for all the items under each scale. The average score for the overall quality of primary care was derived by averaging the values for all scales [15]. The higher the PCAT score, the better a patient’s experience, and the better the quality of primary care. The unmodified Chinese language version of the original simplified PCAT has a reliability coefficient of 0.963, with an acceptable test–retest reliability coefficient of 0.7 (under published).

### Covariates

We collected individual information such as sociodemographic, health service satisfaction, medical-related, and health status characteristics through a self-report questionnaire. Sociodemographic information consisted of age (years), sex (male vs. female), home address, annual family income, and highest education attained. Health status included diabetes and hypertension. Medical-related status included primary type of health insurance and out-of-pocket medical expenditures.

### Statistical analysis

Continuous variables were reported as means ± standard deviations, and relative frequencies were calculated for categorical variables. Welch’s analysis of variance (ANOVA) was used to compare the PCAT scores at different CHCs, and Games-Howell test was used to make multiple comparisons afterwards. We conducted a general linear model (GLM) to estimate the influence of covariates on the PCAT score, including age, sex, family income, education, and medical insurance [16–20]. To figure out the key elements related to health service quality of CHCs, we compared the evaluation items of PCAT and NCQA-PCMH.

Statistical analyses were conducted using SPSS version 21.0 (SPSS Inc., Chicago, IL, USA). All statistical tests were two-sided, and *p*-values < 0.05 were deemed significant.

## Results

### Baseline characteristics at CHCs

A total of 1,776 patients were invited to complete the PCAT questionnaires and 1,744 returned the questionnaire (response rate = 98.2%). After data cleaning, 1,568 valid questionnaires were obtained (effective rate = 89.9%), including 537 in SY, 396 in LH, 149 in LD, 213 in JH, 183 in HL, and 90 in HHG. The baseline characteristics of participants stratified by CHCs are presented in Table 1. Of the 1,568 participants, 55.8% were women and 44.2% were men. Six NCQA-PCMH questionnaires were collected from each community (effective rate = 100.0%).

**Table 1:** Characteristics and PCAT scores of the participants stratified by CHC, n (%)

	HHG (*n* = 90)	HL (*n* = 183)	JH (*n* = 213)	LD (*n* = 149)	LH (*n* = 396)	SY (*n* = 537)	Total (*N* = 1568)
**Age (years)**							
18–25	4 (4.4)	0 (0.0)	0 (0.0)	9 (6.0)	6 (1.5)	1 (0.2)	20 (1.3)
26–30	14 (15.6)	0 (0.0)	0 (0.0)	24 (16.1)	3 (0.8)	14 (2.6)	55 (3.5)
31–40	30 (33.3)	3 (1.6)	33 (15.5)	29 (19.5)	4 (1.0)	28 (5.2)	127 (8.1)
41–50	13 (14.4)	11 (6.0)	40 (18.8)	15 (10.1)	24 (6.1)	27 (6.1)	130 (8.3)
51–60	9 (10.0)	30 (16.4)	34 (16.0)	20 (13.4)	79 (19.9)	79 (14.7)	251 (16.0)
> 60	20 (22.2)	139 (76.0)	106 (49.8)	52 (34.9)	280 (70.7)	388 (72.3)	985 (62.8)
**Sex**							
Male	22 (24.4)	81 (44.3)	78 (36.6)	74 (49.7)	206 (52.0)	232 (43.2)	693 (44.2)
Female	68 (75.6)	102 (55.7)	135 (63.4)	75 (50.3)	190 (48.0)	305 (56.8)	875 (55.8)
**Education**							
Uneducated	1 (1.1)	1 (0.5)	4 (1.9)	5 (3.4)	1 (0.3)	3 (0.6)	15 (1.0)
Primary school	5 (5.6)	16 (8.7)	51 (23.9)	15 (10.1)	69 (17.4)	46 (8.6)	202 (12.9)
Middle school	6 (6.7)	50 (27.3)	78 (36.6)	34 (22.8)	96 (24.2)	144 (26.8)	408 (26.0)
High school	17 (18.9)	88 (48.1)	25 (11.7)	26 (17.4)	146 (36.9)	235 (43.8)	537 (34.2)
≥ College	61 (67.8)	28 (15.3)	55 (25.8)	69 (46.3)	84 (21.2)	109 (20.3)	406 (25.9)
**Annual family income (RMB, yuan)**							
< 100,000	30 (33.3)	58 (31.7)	67 (31.5)	54 (36.2)	54 (36.2)	51 (12.9)	380 (24.2)
100,000–150,000	20 (22.2)	32 (17.5)	59 (27.7)	21 (14.1)	21 (14.1)	112 (28.3)	347 (22.1)
150,000–210,000	24 (26.7)	49 (26.8)	61 (28.6)	27 (18.1)	27 (18.1)	161 (40.7)	434 (27.7)
≥ 210,000	16 (17.8)	44 (24.0)	26 (12.2)	47 (31.5)	47 (31.5)	72 (18.2)	407 (26.0)
**Annual out-of-pocket medical expenditures (RMB, yuan)**							
≤ 800	38 (42.2)	83 (45.4)	51 (23.9)	29 (19.5)	6 (1.5)	151 (28.1)	358 (22.8)
800–1800	16 (17.8)	53 (29.0)	62 (29.1)	24 (16.1)	52 (13.1)	203 (37.8)	410 (26.1)
1800–3000	10 (11.1)	16 (8.7)	22 (10.3)	24 (16.1)	100 (25.3)	69 (12.8)	241 (15.4)
> 3000	26 (28.9)	31 (16.9)	78 (36.6)	72 (48.3)	238 (60.1)	114 (21.2)	559 (35.7)
**Medical insurance**							
Urban resident	9 (10.0)	7 (3.8)	37 (17.4)	39 (26.2)	124 (31.3)	10 (1.9)	226 (14.5)
Employee	71 (78.9)	173 (94.5)	166 (77.9)	105 (70.5)	266 (67.2)	520 (96.8)	1301 (83.0)
Business insurance	10 (11.1)	3 (1.6)	10 (4.7)	5 (3.4)	6 (1.5)	7 (1.3)	41 (2.6)
**Diabetes**							
No	85 (94.4)	128 (69.9)	179 (84.0)	117 (78.5)	264 (66.7)	377 (70.2)	1150 (73.3)
Yes	5 (5.6)	55 (30.1)	34 (16.0)	32 (21.5)	132 (33.3)	160 (29.8)	418 (26.7)
**Hypertension**							
No	71 (78.9)	67 (36.6)	132 (62.0)	85 (57.0)	154 (38.9)	176 (32.8)	685 (43.7)
Yes	19 (21.1)	116 (63.4)	81 (38.0)	64 (43.0)	242 (61.1)	361 (67.2)	883 (56.3)
PCAT scores							
First contact in terms of utilization	2.92 ± 0.75*	3.16 ± 0.53*	3.25 ± 0.57*	3.17 ± 0.59*	2.83 ± 0.57*	3.84 ± 0.31	3.31 ± 0.65
First-contact care in terms of access	2.32 ± 0.57*	2.71 ± 0.52*	2.68 ± 0.49*	2.99 ± 0.51*	2.32 ± 0.45	3.76 ± 0.28	2.97 ± 0.74
Ongoing care	2.13 ± 0.63*	2.87 ± 0.52*	2.71 ± 0.53*	2.91 ± 0.41*	2.46 ± 0.48*	3.96 ± 0.14	3.08 ± 0.78
Coordination of care	2.49 ± 0.58*	2.77 ± 0.49*	3.00 ± 0.55*	3.35 ± 0.56*	2.61 ± 0.54*	3.88 ± 0.29	3.20 ± 0.72
Coordination of information systems	2.66 ± 0.65*	3.22 ± 0.61*	3.38 ± 0.56*	3.30 ± 0.52*	3.22 ± 0.61*	4.00 ± 0.06	3.31 ± 0.74
Comprehensiveness of services available	3.21 ± 0.51*	2.72 ± 0.72*	3.41 ± 0.34*	3.19 ± 0.52*	2.91 ± 0.60*	3.96 ± 0.21	3.36 ± 0.67
Comprehensiveness of services provided	2.75 ± 0.59*	3.02 ± 0.56*	3.35 ± 0.45*	3.08 ± 0.46*	2.51 ± 0.53*	3.91 ± 0.15	3.23 ± 0.70
Family centredness	2.99 ± 0.65*	2.89 ± 0.58*	2.95 ± 0.63*	3.14 ± 0.46*	2.50 ± 0.51*	3.97 ± 0.14	3.20 ± 0.75
Community orientation	2.64 ± 0.71*	2.89 ± 0.46*	2.76 ± 0.55*	3.14 ± 0.45*	2.37 ± 0.52*	3.73 ± 0.34	3.04 ± 0.72
Culturally competent care	2.92 ± 0.63*	2.99 ± 0.69*	3.28 ± 0.59*	2.64 ± 0.64*	2.59 ± 0.56*	3.94 ± 0.20	3.22 ± 0.76
Global PCAT score	2.71 ± 0.40*	2.93 ± 0.43*	3.08 ± 0.33*	3.07 ± 0.35*	2.56 ± 0.29*	3.89 ± 0.13	3.19 ± 0.61

Data are mean (SD) or n (%)

*Abbreviations*: *CHC* Community health centres, *PCAT* Primary Care Assessment Tools

^*^P < 0.05, compared to SY. *P*-value is based on Welch’s analysis of variance (ANOVA)

### Evaluation of patient experiences at CHCs

The PCAT scores of each CHC are also shown in Table 1. PCAT total and sub-domains scores were significantly difference at the six CHCs (*P* < 0.05). Among the six CHCs, Shayuan CHC had the highest PCAT total and sub-domain scores (*P* < 0.05). As a whole, the three dimensions with the highest scores were B, First contact in terms of utilisation (3.31 ± 0.65); F, Coordination of information systems (3.31 ± 0.74); and G, Comprehensiveness of services available (3.36 ± 0.67). The dimensions with the lowest scores were C, First-contact care in terms of access (2.97 ± 0.74); D, Ongoing care (3.08 ± 0.78); and J, Community orientation (3.04 ± 0.72).

### The institutional primary healthcare service quality of CHCs

**Fig. 1:**
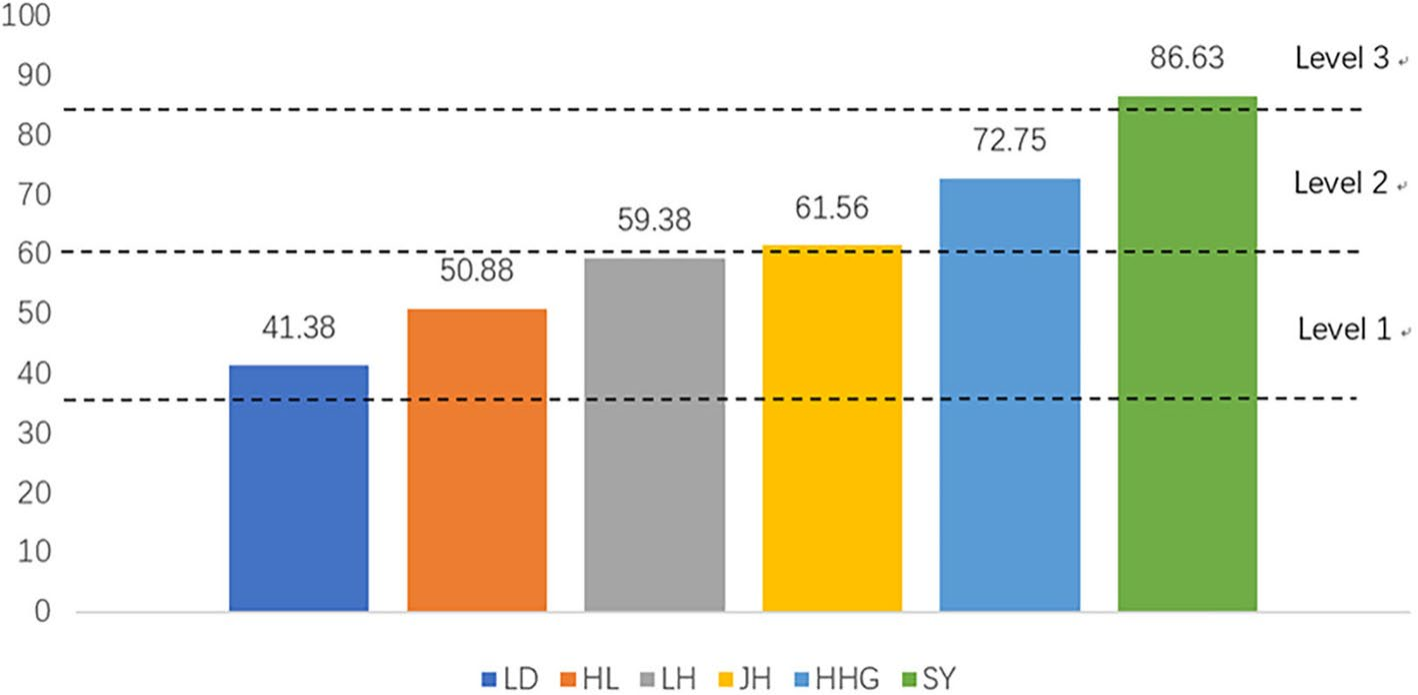
The score of NCQA-PCMH stratified by CHCs

**Fig. 2:**
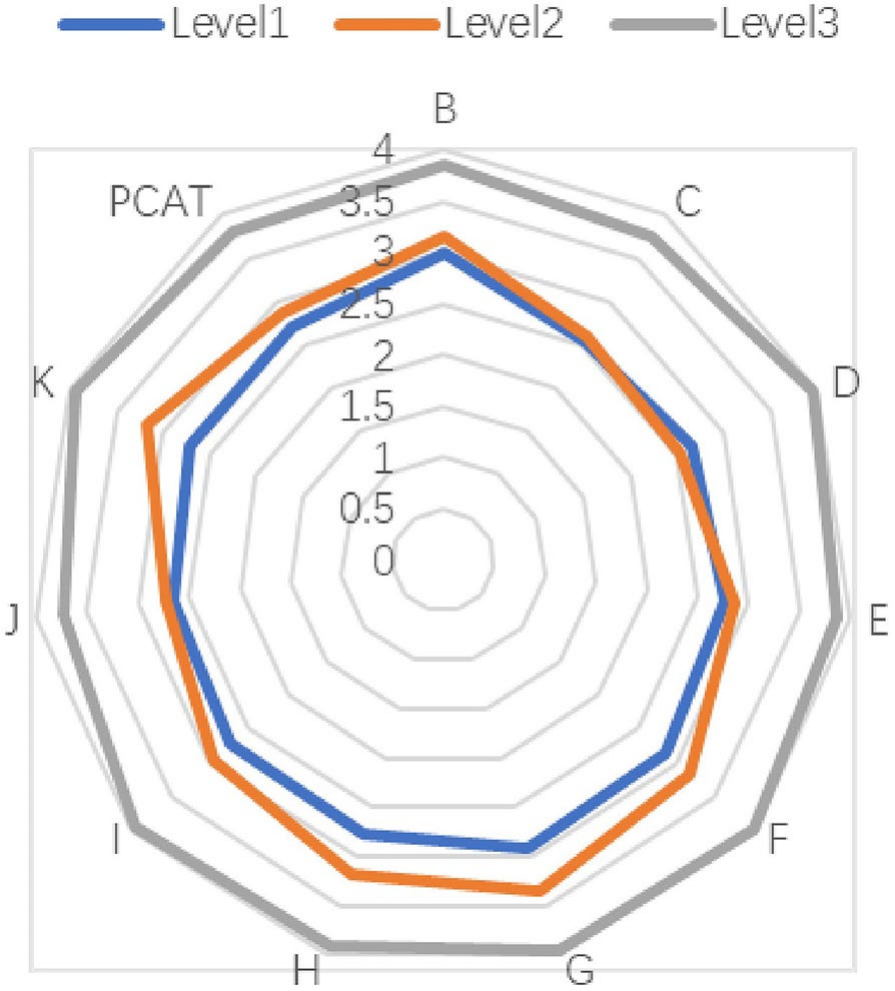
PCAT sub-dimension scores stratified by NCQA-PCMH Level

As shown in Fig. 1 and Table S1, according to the score of NCQA-PCMH, Shayuan CHC had the best service quality as a supplier, while Liede CHC had the poorest service quality. According to the NCQA-PCMH certification grade, Liede (41.38), Hualin (50.88), and Linhe (59.38) were classified as NCQA-PCMH Level 1; Jianghai (61.56) and Huanghuagang (72.75) were Level 2; and Shayuan (86.63) was Level 3. As shown in Fig. 2 and Table 1, the PCAT total and sub-domain scores of Shayuan (SY) CHC, ranked as the Level 3 CHC, were much higher than that of other CHCs (*P* < 0.05).

The scores of each section of the NCQA-PCMH are presented in Table S2. Overall, the two sections with the highest scores were PCMH3, Population health management (14.96) and PCMH6, Performance measurement and quality improvement (13.97). The two sections with the lowest scores were PCMH1, Patient-centred access (8.02) and PCMH2, Team-based care (8.19).

### The key elements related to health service quality of CHCs

As mentioned above, to figure out the key elements related to health service quality of CHCs, we compared the evaluation items of PCAT and NCQA-PCMH. We found that there were many overlapping items between the two evaluation questionnaires (Table S3). For example, both of the scales concern about the accessibility: PCAT-C3 “When your primary care provider (PCP) is open and you get sick, would someone from there see you the same day?” similar to PCMH1-A1 “A1. Providing same-day appointments for routine and urgent care.”; the personal continuity care: PCAT-D1 “When you go to your PCP’s, are you taken care of by the same doctor or nurse each time?” similar to PCMH2-A1 “Assisting patients/families to select a personal clinician and documenting the selection in practice records.”

### The influence of the covariates on PCAT scores

As shown in Table 2, compared to the younger patients, the older patients (> 60 years old) had better patient experiences with higher PCAT scores (*P* < 0.001). Female patients, those with lower educational levels (≤ middle school), or those with employee medical insurance were associated with higher PCAT score, respectively (*P* < 0.05).

**Table 2:** The influence of the covariates on the patients’ experiences

	n(%)	PCAT score (mean ± SD)	β	95%CI	*P* value
**Age (years)**					
≤ 60	583(37.2)	3.09 ± 0.59			
> 60	985(62.8)	3.25 ± 0.62	0.139	0.070 ~ 0.208	< 0.001
**Sex**					
Male	693(44.2)	3.15 ± 0.63			
Female	875(55.8)	3.22 ± 0.60	0.07	0.011 ~ 0.128	0.020
**Education**					
≤ middle school	1160(74.1)	3.22 ± 0.61			
≥ College	406(25.9)	3.08 ± 0.60	-0.106	-0.183 ~ -0.029	0.007
**Annual family income (RMB, yuan)**					
< 150,000	727(46.36)	3.22 ± 0.60			
150,000	841(53.64)	3.16 ± 0.63	-0.058	-0.117 ~ 0.002	0.058
**Medical insurance**					
Urban resident	226(14.4)	2.85 ± 0.45			
Employee	1301(83.0)	3.25 ± 0.62	0.457	0.373 ~ 0.541	< 0.001
Business insurance	41(2.6)	2.97 ± 0.60	0.208	0.011 ~ 0.405	0.039

*Abbreviations*: *CI* Confidence interval, *PCAT* Primary Care Assessment Tools

## Discussion

In this study, we explored the institutional service quality of the CHCs with NCQA-PCMH and the patients’ experiences in the CHCs with PCAT score. Among the six CHCs, Shayuan CHC had the highest PCAT total and sub-domain scores and the highest NCQA-PCMH total and sub-domain scores, as well. Meanwhile, we found that individuals who were older (> 60 years), female, had lower education, or had employee medical insurance reported better patient experiences. The data indicate that CHCs could improve their service quality by addressing patient experiences based on PCAT scales and institutional primary healthcare service quality based on NCQA-PCMH recognition. To our knowledge, this is the first report assessing the primary healthcare service quality of CHCs both with the NCQA-PCMH and the PCAT.

### Service quality of institutions

Of the six CHCs participating in this study, three were NCQA-PCMH Level 1, two were Level 2, and only one was Level 3, indicating that the service quality of most of the institutions in this study still requires improvement, especially concerning patient-centred access and team-based care. Some programmes in the United States have been found to be effective in transforming institutions into higher-level PCMHs. For example, a resident physician cooperation programme helped all 20 participating family medicine residency practices achieve NCQA-PCMH-recognised PCMHs, with 17 attaining Level 3 recognition [21]. NCQA-PCMH recognition may be a helpful metric to improve the institutional primary healthcare service in other countries, including China.

### Patient experiences

In this study, the dimensions with the lowest PCAT scores were dimension C, First-contact care in terms of access; D, Ongoing care; and J, Community orientation. The requirements of these three dimensions are consistent with the responsibilities of family physicians. The World Organization of National Colleges, Academies, and Academic Associations of General Practitioners/Family Physicians notes that family physicians should have six core post-competencies: primary care management, patient-centred care, skills to solve specific clinical problems, comprehensiveness of services available, community-oriented services, and the ability to provide comprehensive services [22]. The Chinese family physician system is still in its infancy. By the end of 2018, the total number of family physicians in Guangzhou was 4,272, with 4.8523 million permanent residents and 1.9759 million key groups [23]. It has been suggested that the signing rate of family physicians in Guangzhou needs improvement. Therefore, the country should vigorously promote the family physician system and improve the signing rate to increase patient satisfaction in the dimensions of C, D, and J. Additionally, expanding the family physician system is an effective way to provide accessible, continuous, and personalised services to patients. The service model of family physicians in China is similar to the American PCMH model, aiming to offer comprehensive, patient-centred, coordinated, accessible, high quality, and safe medical services [9]. It has been suggested that the promotion of family physician contract systems could help CHCs to develop to standardised care for communities, improve the quality of medical services, and enhance patient satisfaction.

### Institutional service quality and patient experiences

Since there was only one CHC, SY CHC, classified as level 3 by NCQA-PCMH, we could not compare the patients’ experiences stratified by NCQA-PCMH levels. However, SY CHC with the highest NCQA-PCMH total and sub-domain scores also had the highest PCAT total and sub-domain scores, which indicates that the facility with the highest recognition scores of NCQA-PCMH provided the most effective primary healthcare service, and that this service quality was recognised by patients. This finding had two distinct implications. First, as health service providers, community health institutions at the meso-level of the health field have a sizeable impact on patient experiences at the micro-level. This is consistent with the Institutional Analysis and Development framework proposed by Ostrom [24]. Therefore, if we want to improve the service experiences of patients, we need to make changes in the meso-level of the health field. For example, referring to the NCQA-PCMH recognition system, the CHCs in China could conduct service quality evaluations and receive level assignments. Then, according to different levels, improvement measures could be put into action to encourage each CHC to become a higher-level CHC. Simultaneously, long-term quality management should be considered. NCQA requires the recognised PCMH to report annually to reconfirm its recognition to determine whether it still maintains excellent service quality [14].

The second implication of these findings is that both the NCQA-PCMH tool and the PACT are valid instruments measuring the performance of primary care. The congruence between performance on one instrument and that on the other instrument shows that both tools are sensitive to the performance of primary care and hence can be used to measure primary care quality at the institutional and patient levels, respectively.

In this study, certain PCAT and NCQA-PCMH metrics evaluating the accessibility of a CHC (PCAT C and PCMH1) revealed comparably low scores, suggesting that both the supply and demand parties perceived accessibility of medical services to be lacking. Thus, the accessibility of medical services is the critical deficiency of CHCs in China. In 2015, a study on the relationship between PCMH practices and access to primary healthcare services showed that patients who called PCMH institutions were more likely to be assigned new appointments and get after-work appointments than patients of non-PCMH institutions, indicating that PCMH-recognised institutions are better at providing access to medical services and providing them in a more timely manner than do their counterparts [25]. This further supports that standard evaluation and recognition of CHCs may be helpful in improving service quality and the patients’ health service experiences. Therefore, China could benefit greatly by introducing the NCQA-PCMH as an institutional primary healthcare service quality assessment tool and ‘the most economic and suitable’ healthcare service model.

Recently, a cross-sectional survey of 1,010 patients’ PCAT scores from China indicated that both urban and rural primary health centres showed a low level of experience with community orientation and family centredness [26]. However, this study did not assess the institutional health quality. In our present study, the scores of PCAT and NCQA-PCMH related to family centredness and community orientation were comparably low, suggesting that the quality of these two elements evaluated by the supply and demand parties were poor. Poor family centredness means doctors seldom make treatment plans with patients or their family members, which could lead to less autonomy and participation from patients in their own medical decisions. According to NCQA-PCMH, primary health service institutions should improve the service quality of Team-Based Care (PCMH2) and Care Management and Support (PCMH4) to provide better family centeredness experiences. Primary health service institutions could provide improve patient experiences by addressing their institutional health service quality according to NCQA-PCMH.

In the present study, we also found that individuals who were older (> 60 years) or female had better patients’ experiences. This was consistent in the key aim and the main functions of CHCs. In China, the main functions of CHCs include prevention, health education, women and children’s care, elderly care, immunization, and physical rehabilitation, all while improving health equity [27]. Thus, lower service quality CHCs could target service improvements to older patients and women to better overall patient experiences.

Although novel in quality evaluation, our study had some limitations; therefore, the results should be interpreted with these in mind. First, as a cross-sectional study, we could not establish a temporal association between process and outcomes. Second, the use of self-reported data might be susceptible to reporting bias, leading to misclassification for some participants. Finally, the sample size was limited because only six CHCs participated. Especially, there is only one CHC in level 3. Thus, we could not estimate the association between PCAT scores and NCQA-PCMH levels.

## Conclusion

Our results indicate that CHCs could improve their service quality by improving both institutional health service quality based on NCQA-PCMH recognition and patient experiences based on PCAT scales. It is thus crucial to establish a service quality evaluation system for primary care institutions in China such as NCQA-PCMH. Further, it is necessary to develop cooperative projects to promote the development of various standardised community programs to improve patients’ primary healthcare service experiences. Our findings can be used to inform future patient-centred primary healthcare policy and management.

## Supplementary Information

**Additional file 1: Figure S1.** Map of the study area and locations of community health centers. **Table S1.** the NCQA-PCMH score for each CHC (x). **Table S2.** The Scores of the NCQA-PCMH Stratified by Levels (x̄ or x). **Table S3.** the relevant items of NCQA-PCMH and PCAT.

## Data Availability

All data generated or analysed during this study are included in this published article and its supplementary information files.

## Abbreviations

CHCs: Community health centres
NCQA-PCMH: National Committee for Quality Assurance Patient-Centred Medical Home
PCAT: Primary Care Assessment Tools
