# Supplementary material for: The institutional primary healthcare service quality and patients’ experiences in Chinese community health centres: results from the Greater Bay Area study, China
Source: Int J Equity Health. 2021 Aug 30;20:198. doi: 10.1186/s12939-021-01538-8 (PMC8404182; doi:10.1186/s12939-021-01538-8)
Supplement: Supplementary file 1 — Additional file 1: Figure S1. Map of the study area and locations of community health centers. Table S1. the NCQA-PCMH score for each CHC (x). Table S2. The Scores of the NCQA-PCMH Stratified by Levels (x̄ or x). Table S3. the relevant items of NCQA-PCMH and PCAT. [file 12939_2021_1538_MOESM1_ESM.docx]

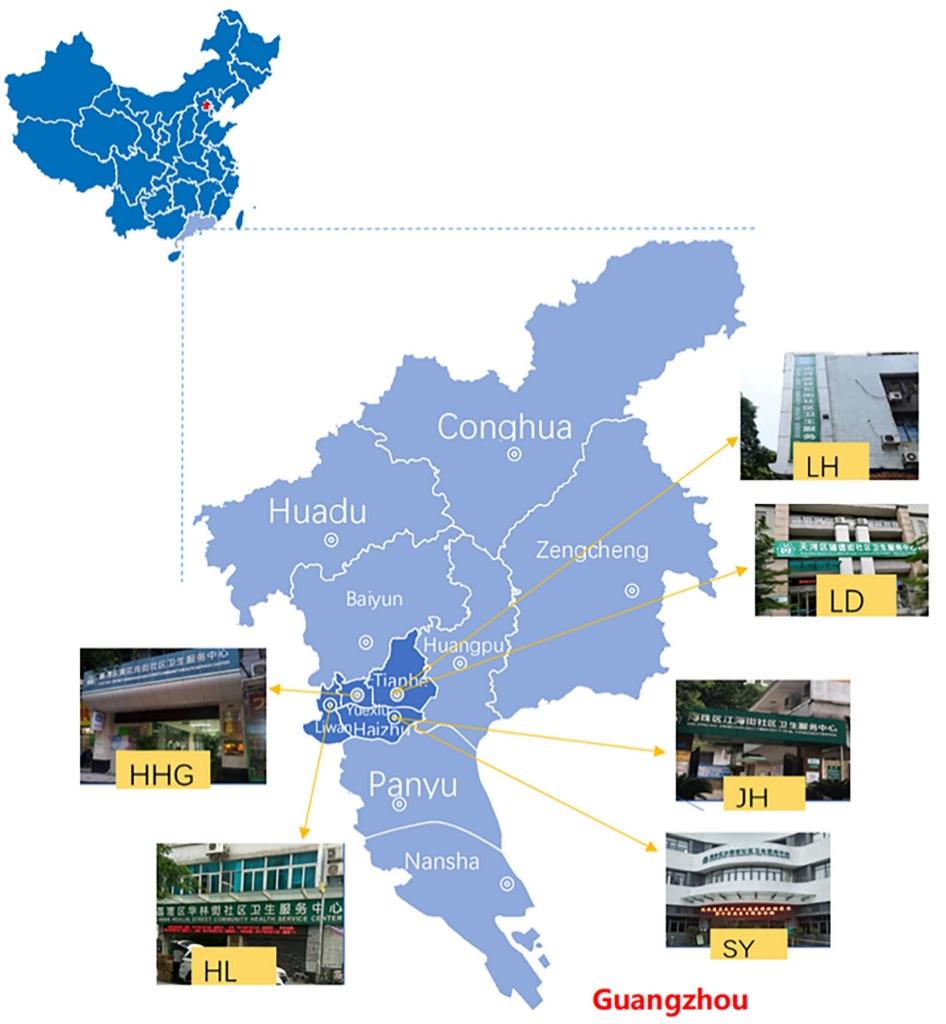


Figure S1 Map of the study area and locations of community health centers

Table S1 the NCQA-PCMH score for each CHC (x)

|  | HHG | | HL | | JH | | LD | LH | SY |
| --- | --- | --- | --- | --- | --- | --- | --- | --- | --- |
| PCMH1: Patient-centred access | 7.00 | 7.25 | | 7.62 | | 8.00 | | 6.50 | 9.50 |
| PCMH2: Team-based care | 5.25 | 6.88 | | 7.88 | | 7.13 | | 5.88 | 11.38 |
| PCMH3: Population health management | 12.75 | 13.75 | | 15.00 | | 12.00 | | 13.25 | 18.00 |
| PCMH4: Care management and support | 19.00 | 12.50 | | 8.25 | | 11.25 | | 10.00 | 14.00 |
| PCMH5: Care coordination and care transitions | 10.50 | 0 | | 7.56 | | 1.50 | | 12.00 | 16.50 |
| PCMH6: Performance measurement and quality improvement | 18.25 | 10.50 | | 15.25 | | 1.50 | | 11.75 | 17.25 |
| Total score | 72.75 | 50.88 | | 61.56 | | 41.38 | | 59.38 | 86.63 |

NCQA-PCMH: National Committee for Quality Assurance Patient-Centred Medical Home

Table S2 The Scores of the NCQA-PCMH Stratified by Levels (x̄ or x)

|  | | Level 1  (n=3) | Level 2  (n=2) | Level 3  (n=1) | Total  (n=6) |
| --- | --- | --- | --- | --- | --- |
| PCMH1: Patient-centred access | 7.25 | | 7.31 | 9.50 | 8.02 |
| PCMH2: Team-based care | 6.63 | | 6.56 | 11.38 | 8.19 |
| PCMH3: Population health management | 13.00 | | 13.88 | 18.00 | 14.96 |
| PCMH4: Care management and support | 10.08 | | 13.63 | 14.00 | 12.57 |
| PCMH5: Care coordination and care transitions | 4.50 | | 9.03 | 16.50 | 10.01 |
| PCMH6: Performance measurement and quality improvement | 7.92 | | 16.75 | 17.25 | 13.97 |
| Total score | 50.54 | | 67.16 | 86.63 | 68.11 |

NCQA-PCMH: National Committee for Quality Assurance Patient-Centred Medical Home

Table S3 the relevant items of NCQA-PCMH and PCAT

| Contact-access | PCMH | PCMH1. Patient-Centered Access | A1. Providing same-day appointments for routine and urgent care. |
| --- | --- | --- | --- |
|  |  |  | A4. Availability of appointments. |
|  | PCAT | C First Contact-Access | C3. When your PCP is open and you get sick, would someone from there see you the same day? |
| Outside the clinic | PCMH | PCMH1. Patient-Centered Access | A3. Providing alternative types of clinical encounters. |
|  |  |  | B2. Providing timely clinical advice by telephone. |
|  |  |  | C2. The capability to view, download or transmit their health information to a third party. |
|  | PCAT | C First Contact-Access | C4. When your PCP is open, can you get advice quickly over the phone if you need it? |
|  |  | D Ongoing Care | D4. If you have a question, can you call and talk to the doctor or nurse who knows you best? |
| Case information preservation | PCMH | PCMH1. Patient-Centered Access | B1. Providing continuity of medical record information for care and advice when office is closed. |
|  | PCAT | F Coordination (Information Systems) | F3. When you go to your doctor, is your medical record always available? |
| Patients themselves are aware of personal health information | PCMH | PCMH1. Patient-Centered Access | C1. More than 50 percent of patients have timely access to their health information. |
|  |  |  | C3. Clinical summaries are provided to patients/families/caregivers upon request. |
|  |  | PCMH3. Population Health Management | Element A - Patient Information |
|  |  |  | Element B - Clinical Data |
|  | PCAT | F Coordination (Information Systems) | F2. Could you look at your medical record if you wanted to? |
|  |  |  | F3. When you go to your PCP, is your medical record always available? |
| Personal continuity care | PCMH | PCMH2. Team-Based Care | A1. Assisting patients/families to select a personal clinician and documenting the selection in practice records. |
|  | PCAT | D Ongoing Care | D1. When you go to your PCP’s, are you taken care of by the same doctor or nurse each time? |
| Family-centeredness | PCMH | PCMH2. Team-Based Care | A4. Collaborating with the patient/family to develop/implement a written care plan for transitioning from pediatric care to adult care. |
|  |  | PCMH4. Care Management and Support | B5. Is provided in writing to the patient/family/caregiver. |
|  | PCAT | I Family-Centeredness | I1. Does your PCP ask you about your ideas and opinions when planning treatment and care for you or a family member? |
| Referral Coordination | PCMH | PCMH2. Team-Based Care | B1. The practice is responsible for coordinating patient care across multiple settings. |
|  |  |  | B8. Instructions on transferring records to the practice, including a point of contact at the practice. |
|  |  | PCMH5. Care Coordination and Care Transitions | B5. Gives the consultant or specialist the clinical question, the required timing and the type of referral. |
|  |  |  | B7. Has the capacity for electronic exchange of key clinical information and provides an electronic summary of care record to another provider for more than 10 percent of referrals. |
|  |  |  | B9. Documents co-management arrangements in the patient medical record. |
|  |  |  | B10. Asks patients/families about self-referrals and requesting reports from clinicians. |
|  |  |  | C2. Shares clinical information with admitting hospitals and emergency departments. |
|  |  |  | C3. Consistently obtains patient discharge summaries from the hospital and other facilities. |
|  |  |  | C7. Exchanges key clinical information with facilities and provides an electronic summary-of-care record to another care facility for more than 10 percent of patient transitions of care. |
|  | PCAT | E Coordination | E8. Did your PCP discuss with you different places you could have gone to get help with that problem? |
|  |  |  | E9. Did your PCP or someone working with your PCP help you make the appointment for that visit? |
|  |  |  | E10. Did your PCP write down any information for the specialist about the reason for the visit? |
|  |  |  | E12. After you went to the specialist or special service, did your PCP talk with you about what happened at the visit? |
|  |  | F Coordination (Information Systems) | F1. When you go to your PCP, do you bring any of your own medical records, such as shot records or reports of medical care you had in the past? |
| Language services | PCMH | PCMH2. Team-Based Care | ELEMENT C - Culturally and Linguistically Appropriate Services |
|  | PCAT | K Culturally Competent | K2. Would you recommend your PCP to someone who does not speak English well? |
| Comprehensive health care | PCMH | PCMH2. Team-Based Care | D6Training and assigning members of the care team to support patients/families/caregivers in self-management, self-efficacy and behavior change. |
|  |  | PCMH4. Care Management and Support | A1. Behavioral health conditions. |
|  |  |  | B1. Incorporates patient preferences and functional/lifestyle goals. |
|  |  |  | E6. Maintains a current resource list on five topics or key community service areas of importance to the patient population including services offered outside the practice and its affiliates. |
|  | PCAT | D Ongoing Care | D7. Does your PCP know you very well as a person, rather than as someone with a medical problem? |
|  |  |  | D9. Does your PCP know what problems are most important to you? |
|  |  | J Community orientation | J1. Does anyone at your PCP’s office ever make home visits? |
| Patient engagement | PCMH | PCMH2. Team-Based Care | D10. Involving patients/families/caregivers in quality improvement activities or on the practice advisory council. |
|  | PCAT | J Community orientation | J3. Does your PCP get opinions and ideas from people that will help to provide better health care? |
| Drug use follow-up | PCMH | PCMH3. Population Health Management | D5. Medication monitoring or alert. |
|  |  | PCMH4. Care Management and Support | ELEMENT C - Medication Management |
|  |  |  | C1. Reviews and reconciles medications for more than 50 percent of patients received from care transitions. |
|  |  |  | C2. Reviews and reconciles medications with patients/families for more than 80 percent of care transitions. |
|  |  |  | C3. Provides information about new prescriptions to more than 80 percent of patients/families/caregivers. |
|  |  |  | C4. Assesses understanding of medications for more than 50 percent of patients/families/caregivers, and dates the assessment. |
|  |  |  | C6. Assesses response to medications and barriers to adherence for more than 50 percent of patients, and dates the assessment. |
|  | PCAT | H Comprehensiveness (Services Provided) | H7. Checking on and discussing the medications you are taking |
| Immunization | PCMH | PCMH3. Population Health Management | C1. Age- and-gender appropriate immunizations and screenings. |
|  | PCAT | G Comprehensiveness (Services Available) | G2. Immunizations (shots) |

PCAT: the Primary Care Assessment Tools; PCMH: Patient-Center Medical Home.
